# Implementing the Additional Roles Reimbursement Scheme in seven English Primary Care Networks: a qualitative study

**DOI:** 10.3399/BJGP.2023.0216

**Published:** 2024-04-16

**Authors:** Donna Bramwell, Jonathan Hammond, Lynsey Warwick-Giles, Simon Bailey, Kath Checkland

**Affiliations:** Centre for Primary Care and Health Services Research, University of Manchester, Manchester.; Centre for Primary Care and Health Services Research, University of Manchester, Manchester.; Centre for Primary Care and Health Services Research, University of Manchester, Manchester.; Centre for Health Services Studies, University of Kent, Canterbury, Kent.; Centre for Primary Care and Health Services Research, University of Manchester, Manchester.

**Keywords:** additional roles reimbursement scheme, primary health care, primary care networks, qualitative research, workforce

## Abstract

**Background:**

The Additional Roles Reimbursement Scheme (ARRS) provides funding to Primary Care Networks (PCNs) in England to recruit additional staff into specified roles. The intention was to support general practice by recruiting an extra 26 000 staff by 2024, increasing access and easing workload pressures.

**Aim:**

To explore the establishment of the ARRS as part of PCNs’ development to understand their role in supporting general practice.

**Design and setting:**

A longitudinal, qualitative case study involving seven geographically dispersed PCNs across England.

**Method:**

Data were collected from July 2020 to March 2022, including 91 semi-structured interviews and 87 h of meeting observations. Transcripts were analysed using the framework approach.

**Results:**

Implementation of the ARRS was variable across the study sites, but most shared similar experiences and concerns. The COVID-19 pandemic had a significant impact on the introduction of the new roles, and significant variability was found in modes of employment. Cross-cutting issues included: the need for additional space to accommodate new staff; the inflexibility of aspects of the scheme, including reinvestment of unspent funds; and the need for support and oversight of employed staff. Perceived benefits of the ARRS include improved patient care and the potential to save GP time.

**Conclusion:**

The findings suggest the ARRS has potential to fulfil its objective of supporting and improving access to general practice. However, attention to operational requirements including appropriate funding, estates, and management of staff is important if this is to be realised, as is clarity for the scheme post-contract end in 2024.

## Introduction

Primary Care Networks (PCNs) were established in 2019 and are collaborations of general practices in the English NHS, the majority of which cover populations of approximately 30 000–50 000.^1^^,^^2^ PCNs were introduced as a mechanism by which to address the increasing healthcare pressure on primary care, with the intention that interpractice collaboration will allow economies of scale in providing integrated, coordinated care for patients within their neighbourhood.

Practices are incentivised to work together through the Network Contract Directed Enhanced Service (DES),^3^ which is an addition to the standard GP contract. Participation is voluntary, and, among other things, the Network Contract DES provides reimbursement for PCNs to recruit new staff for a variety of clinical and non-clinical roles, with the aim of helping to solve workforce shortages in general practice and reduce workload pressures.^1^ Known as the Additional Roles Reimbursement Scheme (ARRS), the extra staff are intended to take on some of the tasks of GPs, freeing up their time, and also improving access to general practice.

The ARRS is a nationwide scheme, initially planned to run for 5 years across England but has now been extended.^1^ The early goal was to recruit 20 000 full-time equivalent staff by 2024, which has since been revised to a target of 26 000 staff.^4^ The contract states that these roles must be additional, rather than filling existing vacancies. The first roles agreed by NHS England for recruitment into primary care are in the left-hand column in Box 1; it was argued by NHS England that these roles were chosen because they could make a demonstrable impact on the workload of general practice.^1^ Additional new roles have been added over time, as shown in the right-hand column in Box 1.

**Box 1.** Titles of additional roles

**Job titles, 2019/2020 — target recruitment 20 000 staff^2^**	**Job titles, 2021/2024 — target recruitment 26 000 staff^4^**
Clinical pharmacists (2019) Social prescribing link workers (2019) First contact physiotherapists Physician associates	Pharmacy technicians Care coordinators Health and wellbeing coaches Dietitians Podiatrists Occupational therapists Community paramedics Mental health professionals Nurse associates and trainee nurse associates Advanced practitioners General practice assistants (September 2022) Digital transformation leads (September 2022) Adult mental health practitioners (2022) Children and young people mental health practitioners (2022)

Despite the fact that the scheme is now well established there has been little empirical research on it to date. A report by Baird *et al* describes multiple factors impinging on the successful implementation of the roles, including challenges with the lack of management and human resources support, and achieving multidisciplinary working.^5^ Baird *et al* also highlight confusion as to whether these roles were intended to deliver new services or to undertake core general practice work. Opinion pieces on the scheme, such as that by Khan, focus on how the roles are perceived by patients, calling for clarity around nomenclature and standardised practices for the roles across the board.^6^ Abrams and Eaton argued for role clarity but also mentioned that the new roles put extra strain on GPs in terms of supervision, adding to, rather than alleviating, pressures.^7^

**Table:** How this fits in

The Additional Roles Reimbursement Scheme (ARRS) was introduced into general practice as a way of funding extra staff to help address challenges faced by primary care in England. Since inception in 2019, the scheme has been subject to little empirical research but what is available points to the complexities of introducing a mix of skills and new staff into the general practice environment, and suggests that any such large-scale change is complex to manage and to operationalise. The current research focused on those factors that need to be addressed to support successful integration of staff, and explored the implications for future policy and the ability of the ARRS to fulfil its potential.

In the current study, the findings from a longitudinal study examining the development of PCNs are reported. Although not the only focus of the study, the ARRS comprised a large subset of the data discussed in this study. The focus is on the experiences of implementing the ARRS from a range of perspectives. Three broad themes are addressed:

- the organisation of the ARRS;
- recruitment; and
- the roles themselves.

These findings are considered in the light of existing literature about the changes to skill mixing in primary care, and it concludes with a consideration of the factors affecting the potential of the ARRS to address the intended aim of supporting general practice and addressing challenges arising out of workforce shortages.^1^

## Method

This study reports on data collection from work package 3 of a larger, longitudinal mixed-methods study, and represents the qualitative element of the study conducted between July 2020 to March 2022. A case study design was chosen to allow us to compare and contrast across sites. Seven PCN sites across England were identified and recruited based on data from initial telephone interviews with clinical commissioning group (CCG) staff.

A total of 91 semi-structured interviews were conducted, which were augmented with 87 h of meeting observations (these included key PCN meetings such as clinical director meetings and PCN board/strategy meetings to which the researchers were invited to observe — usually by the clinical director — with the consent of all participants). Case study sites were selected for heterogeneity, including size, population demographics, and geographical location (Table 1). This reflects the wide diversity of PCNs as described by Morciano *et al*^8^ in their analysis of all 1250 PCNs in England.

**Table 1.** Case study demographics

**Characteristic**	**PCN A**	**PCN B2**	**PCN B3**	**PCN C**	**PCN D**	**PCN E1**	**PCN E2**
**GP member practices,*n***	10–15	15–20	10–15	5–10	5–10	5–10	5–10
**Rurality, approximate %**	0.5	1.5	0.1	0.1	>20.0	2.0	1.0
**Patient population, *n***	60 000–70 000	90 000–100 000	70 000–80 000	50 000–60 000	80 000–90 000	30 000–40 000	30 000–40 000
**Population deprivation**	High	Mixed	High	Mixed	Mixed	High	High

*PCN = Primary Care Network.* PCNs were labelled for anonymity. Site B1 originally agreed to participate but did not respond further.

CCGs were contacted by email to invite them to participate and to identify key contacts. Within each site, sampling of participants was both purposive, including staff expected to have direct knowledge of PCN development, and snowballing, asking each interviewee to identify others who may have a relevant perspective. Potential participants were provided with information about the study and invited to take part in an interview. No participants who were invited declined to take part in the study.

Data collection was conducted by four experienced researchers; two female (the first and third authors) and two male (the second and fourth author). As a result of COVID-19 pandemic restrictions, interviews were carried out remotely via the Microsoft Teams or Zoom online platforms, involved only the researcher and participant, and lasted approximately 1 h.

Interviewees included PCN clinical directors, managers, leadership team members, GP members, ARRS staff, CCG staff, commissioners, and staff from other local providers including third-sector organisations, NHS trusts, and local authorities. Interview guides (see Supplementary Information S1–S3) were initially based on the research question, relevant literature, policy documentation, and existing knowledge of primary care, and were refined over time to incorporate insights from ongoing analysis. Meeting observations lasted an average of 2 h. Audio-recorded interviews (with consent) were transcribed verbatim by a specialist transcription company and were not returned to the participants for checking. Post-interview and meeting field notes were documented and typed up by the researchers. Data collection continued until the team agreed that no new lines of analysis were developing.

### Analysis

Analysis of both transcribed interviews and field notes was undertaken iteratively by the team members (the first four authors). Participants were assigned an ID code (Nxxx) to ensure anonymity, and anonymised interview transcripts and field notes were imported into qualitative data analysis programme NVivo (version 12 Plus) and analysed using the Framework Method.^9^ This is a systematic and structured way of managing textual data analysis whereby text is synthesised and data are ‘charted’ into a matrix to identify, define, interpret, and ultimately explain themes, concepts, and associations across and between the data sources.

A coding framework was developed by the research team (the first four authors), using both inductive and deductive approaches. Initial a priori codes were developed from the research aims and literature, and new codes were developed iteratively during analysis. These were discussed among the team. The framework was applied to the coding of each transcript, allowing comparison between interviews and field notes. Coding was discussed at regular research team meetings to ensure consistency. Codes relating to the ARRS were extracted and a second-order thematic analysis applied, the results of which are provided here.

## Results

Many participants commented on the benefits of the scheme and the support that ARRS staff could provide. However, experiences varied, with some PCNs finding it easier to incorporate the roles than others. The current study presents the authors’ cross-case analysis of the interviews and meeting observations combined. Using both interviews and meeting observations provided a more holistic picture of the developing PCN and ARRS landscape than utilising one approach alone. Data gathered from interviews gave more nuanced perspectives of the scheme ‘on the ground’ when compared with higher-level discussions observed in meetings. Both approaches thus complement each other, and when analysed in combination highlight a wide variation in how the ARRS was being operationalised within and across PCNs, and the factors identifiable as supporting or hindering this.

### Organisation of ARRS

PCNs are not formal organisations (although some have decided to form a limited company) and therefore staff cannot be employed directly by the network. Each PCN must therefore make decisions about how the scheme is to be operationalised; this could be challenging.

#### Employment models

A number of employment models and modes of contracting for ARRS staff and services were found to be in use across the seven PCNs:

- contracted through an agency, so staff are independent contractors;
- a deployment model, with community health trusts or acute hospital trusts holding the employment contract and subcontracting staff to provide services in PCN practices;
- subcontracting through third-sector organisations;
- employed through another legal entity such as a legally constituted GP federation; and
- employed through a single-lead GP member practice, or a distributed model with different roles employed by different practices.

Reasons for choosing particular models varied, but the current study found some preference for approaches that limited employment risks to individual GP practices, such as by using staff employed through a local hospital or community trust. One participant described the challenges in managing contractual models as a *‘minefield’* (N0303t, head of primary care). Perceptions of risks associated with employing staff directly were voiced by many sites, including performance management, administration, and administrative liabilities such as pensions, sick pay, or redundancy. This created complexities regarding the degree of control networks had over additional role (AR) staff. An example cited was that NHS frontline staff were required to be COVID-19 vaccinated^10^ but PCNs could not enforce this for frontline voluntary-sector staff such as social prescribers.

One interviewee suggested that the reluctance to employ staff directly was underpinned by concern about the longevity of PCNs:

*‘And if there’s a big system change, then all of a sudden, you’ve got, you know, ten employed people, for whom you need to either find jobs or find redundancy, and we don’t want to get into that.’*

(N64019, clinical director)

A further area of variation was found in how staff were used, with some evidence of disagreements between practices. For example, the remit of the care coordinator role varied across the sites in the current study, from purely administrative roles through to working with frequently attending patients and care home coordination:

*‘And from discussion with the staff that I’ve spoken to there, there’s a very different approach in each PCN as to what they’re going to ask of their ARRS worker when they’re provided.’*

(N111qm, care coordinator)

#### Funding

ARRS funds represent a significant proportion (over 50%) of the investment associated with PCNs. However, using the funding was not necessarily straightforward because of relatively rigid rules dictating that it can only be spent on staff salaries. Moreover, there was a significant underspend during the first 2 years of the scheme,^11^ but the unused funding could not be used in other ways, such as alternative ways of investing in patient care, employing staff outside the prescribed roles, or on other priorities such as improving estates. This caused some resentment, as observed in PCN meetings and as the following quote suggests:

*‘… if you’re trying to grow but you really haven’t got any possibility because of space, you can redirect some of the ARRS funding to estates to help to fund and therefore grow your workforce, there’s ways of means of doing this. What you end up with is at the end of the year a massive pot of the ARRS funding that’s not been used and it doesn’t make any sense when part of the reason … probably the main reason it’s not been used is because people can’t put anyone anywhere.’*

(N190n7, finance manager)

This initial underspend was also driven by recruitment difficulties exacerbated by the pandemic and that reimbursement for salaries was initially only 70%. Some practices were reluctant to employ staff when needing to find the additional 30% because, as one participant said, *‘they didn’t know where that was going to come from’* (N130iy, pharmacy manager).

Although later funding was intended to cover the full salary of staff employed, a few PCNs told us that there could still be a shortfall, particularly around costs for sick and maternity pay, and to cover pensions and National Insurance increases. Employing through an agency or other third party did not necessarily alleviate these problems, as they may be reflected in additional fees and expenses to be paid by the PCN.

#### Estates

There were considerable frustrations within case study sites regarding the lack of available space for extra staff. The majority of those interviewed, from GPs through to clinical directors, commented on the lack of a suitable room for the AR staff, and the AR staff themselves told us this was a problem. This was so significant in some areas as to be seen as a potential obstacle to the success of PCNs:

*‘The one point I would really, really stress is the estates. So if you’re going to take anything out of this, it maybe needs to be raised at a certain level, the estates needs considering … We’ve got funding for* [staff]*, but we haven’t considered where we might put people. It’s a bit of an oversight that every single PCN I’ve spoken to seems to be struggling with.’*

(N190n7, finance manager)

The lack of space not only influences individual job satisfaction, it may also have important effects on relationships, the extent to which staff feel part of a team, and their sense of belonging to the practice, as one coordinator illustrated:

*‘So we’re hoping that our hub should be set up and running then ... so we’ll have a big space, big area, store all our stuff. So that will make a difference as well. We can come together as a team then. Even if it’s just once now and then, all of us together rather than just passing by’.*

(N3112e, care coordinator)

### Recruitment

Recruitment of ARs across the sites was variable, with all of the PCNs reporting initial difficulties in recruiting and retaining staff to some extent. Challenges were specific to particular roles in each site. The employment of pharmacists in primary care, for example, has been a long-standing trend making their employment and deployment easier to manage, whereas other, newer roles such as physician associates could be more difficult. Participants also discussed that decisions about which types of staff to recruit were often driven by the availability of funds for particular roles or the availability of staff to recruit, rather than by an assessment of local staffing needs.

Furthermore, all PCNs were initially recruiting from a fixed pool of available staff for a limited number of roles. This was especially notable in the first year of the scheme where contractual restrictions only allowed PCNs to recruit two specific roles. PCNs were therefore competing for staff:

*‘So I know about our PCN, we mainly recruited pharmacists, we’ve gone down that model of having a large number of pharmacists. And there were difficulties in recruiting, sort of, and one of the things that we saw was that ’cause there was so many jobs out there, you know, we would interview, offer the places and then they would go elsewhere. Or they would accept and then end up not taking the offer ’cause they were being offered something elsewhere that was for more money etcetera.’*

(N380bn, primary care lead)

COVID-19 had a significant impact on both the recruitment and deployment of AR staff. Lockdowns affected both the ability to recruit and the experience of staff recruited, who often did not meet other team members.

### Roles — perceptions, integration, and support

PCN members and staff were generally very positive about the potential of the ARRS to support general practice, summed up in this quote:

*‘When we have the conversations about putting in additional roles in the PCN I think that’s where I get excited and passionate and feel we’re really doing something worthwhile here for the population. So I’d like to see the development of the additional roles work.’*

(N430hm, practice nurse)

Comments on the benefits of the scheme were wide ranging including having the ability to delegate tasks, being able to signpost patients for relevant non-clinical services, and reduce pressure on GPs’ time, as this quote suggests:

*‘So, it just builds that non-clinical capacity in the team and hopefully might divert some of that demand because the target is the high users of GP services initially.’*

(N08067, community development)

Further, the introduction of early intervention services, such as an initiative in one of the PCNs of a multidisciplinary team to provide a light-touch, biopsychosocial approach to mental health care, was seen as a way to hopefully prevent the need for patients to access both primary and secondary care.

However, incorporating new staff members into teams is not always initially straightforward, especially if their time is split between sites. Some AR staff expressed feelings of isolation and not being valued or welcomed as part of the team. This could be because of lack of space or misunderstandings about their role. The authors of the current study found that understandings of the different roles could also be different across PCNs, which is not helped by a lack of common nomenclature to describe roles’ tasks and titles.

Some participants also mentioned the lack of communication about what staff are available, what they do, and how they can benefit the practice, as this PCN manager illustrated:

*‘They are quite new really, we haven’t recruited too many in for various different reasons, but one practice they’ve been quite advanced and being aware of some of the roles and utilising them. So it’s also about, I guess, making sure that everyone is informed around the roles and understanding what the roles do and how they can be utilised.’*

(N1503y, PCN manager)

These complexities could have a negative impact on AR staff themselves, and the authors of the current study heard of staff leaving because they did not feel part of a team or felt that their professional role was not being utilised appropriately. Interviewees highlighted the importance of paying attention to the support needed by new staff and the need for professional development:

*‘You know, it’s like there needs to be … seniority needs to be taken into consideration with all the ARRS roles, to give people room to develop … Because you need development opportunities for staff to keep them keen.’*

(N011c6, PCN manager)

Finally, some interviewees suggested that GPs lack the time they need to supervise and embed AR staff into multidisciplinary teams and practices. Staff who have not worked in general practice before can require considerable training, and this was a further drain on GPs’ time.

## Discussion

### Summary

The ARRS represents a significant investment in primary care staff, supporting a large-scale influx of new staff. Any such large-scale change is likely to be complex to manage and operationalise. The current study suggests widespread support for the scheme, but also highlights a number of significant complexities around its implementation that could be addressed in policy. The employment of staff is complex, with multiple different employment models in use, often within the same PCN for different categories of staff. Each way of employing staff brings with it different requirements with regard to support, oversight, and management.

This complexity was exacerbated at the time by concerns about would happen after the 5 years of the initial contract elapsed, although subsequent statements from NHS England suggested that, whatever happened to GP contracts more widely, ARRS funding would be protected and continued^12^, as is the case.^13^

Considerable heterogeneity was found across the case study sites, but a number of cross-cutting issues were evident. These include: the need for additional investment in estates to provide accommodation for additional staff; the inflexibility of current funding, particularly around the use of underspends; and the complexities associated with embedding and supporting staff to ensure retention and job satisfaction. Considerable GP time was required for training and ongoing supervision, potentially limiting the extent to which the scheme will reduce GP workload.

### Strengths and limitations

The longitudinal, case-study, and multimethod approach made it possible to capture a wide range of perspectives and experiences of the ARRS across time. The COVID-19 pandemic inevitably caused issues both for the scheme and for this research. The authors were able to quickly pivot to remote data-collection methods, but the need to redeploy staff to meet the demands of the pandemic response meant that many intended PCN service delivery activities as part of the Network Contract DES were postponed or altered, with consequences for the ARRS. As this study was conducted in the early stages of PCN development, the findings therefore focus on the initial operationalisation of the scheme. A number of policy changes since then have broadened the scope of the scheme, with new roles added. Further research is required to understand in more depth how the initial issues that the authors have identified in the current study play out in the longer term, with particular emphasis on how new workers are being integrated into practice teams and managed, as well as quantitative study of their impact on workload.

### Comparison with existing literature

There has been little empirical work specifically addressing the ARRS. Baird *et al* undertook four focus groups and interviews with a total of 48 responders, spanning four ARs, PCN directors, and some national stakeholders in late 2021.^5^ As well as some confusion around the remit and purpose of the employment of AR staff, their report highlighted the need for staff to be adequately supported to allow integration into practice teams. This finding is confirmed in the current study, with AR staff reporting some difficulties, particularly where they were working across several practices. In keeping with the current study, they also highlight challenges with estates, a lack of certainty over long-term funding, and the need for a clearer vision of the contribution that the roles can make as key issues for the future. Other commentators similarly^6^^,^^7^ note the need for clarity around the capabilities associated with particular roles, and consistency in how this is achieved and named. The current findings also resonate with those of a recent study exploring the role of nine social prescribing link workers, which demonstrated that there was ambiguity around the definition and remit of this complex role, and that better understanding, support, and training for these roles were required.^14^

Moving beyond the specificities of the ARRS, a number of authors have studied skill-mix change in primary care more generally.^15^^,^^16^ A large-scale mixed-methods study of the changes to mixing skills in primary care^17^ found that the integration of new staff required complex processes to match patients’ problems to staff with the appropriate capabilities, and appropriate flexibility to cope with inevitable instances where staff were unable to adequately deal with particular patients. This could result in significant inefficiencies, as patients sometimes needed additional appointments. The current study suggests that these types of difficulties may be multiplied in situations where new staff only spend a limited part of each week in any one practice. In addition, McDermott *et al* found new types of workers required significant supervision by GPs, limiting the ability for their employment to free up GP time, and the current study suggests that this also applies to AR staff.^17^

More generally, commentators have questioned whether or not the employment of different types of clinical staff will necessarily reduce GP workload.^18^^,^^19^ The current study was not able to directly address this question, in part because of the unique circumstances associated with the COVID-19 pandemic. However, it does point to some specific issues around supervision of new staff that may be relevant to this question.

### Implications for research and practice

The authors’ early research among stakeholders identified supporting general practice as one of the main policy objectives underlying PCNs, and the provision of funding to employ new staff through the ARRS was seen as an important mechanism by which this objective would be realised.^20^ This follow-up research highlights a number of policy-relevant issues that may need to be addressed if this is to be achieved.

First, the initial contract was for 5 years and due to finish this year. Clearly the pandemic was an unforeseen and unwelcome intrusion that materially affected the early stages of the ARRS. Nevertheless, lack of certainty over the funding stream was an important element in determining how practices and PCNs operationalised the scheme at the time.

Second, the provision of adequate estates within which to house additional staff is clearly important. Current policy does not include capital funding and should ARRS funding continue in the future then it may become necessary to consider how additional building capacity could be financed and obtained.

Third, current formulae for funding ARRs only partially account for deprivation.^21^ In order to avoid exacerbating existing maldistribution of staff related to need (the so-called ‘inverse care law’^22^) future iterations of the funding could be adjusted more comprehensively to reflect deprivation.

Finally, the current study suggests that in the longer term, support for human resources processes and good employment practices would be useful in ensuring that staff who move into community roles are not disadvantaged, and that PCNs have adequate performance monitoring and oversight procedures as new types of staff take on a wide variety of roles.

In conclusion, the current study suggests that the ARRS has the potential to fulfil its objective of supporting and improving access to general practice. However, a number of factors were identified that need to be taken into account in further iterations of the scheme, including: the need for flexible funding models; lack of accommodation in the current primary care estate; and attention to the management and oversight of staff. Greater focus on these is suggested if policy aims are to be realised, as is clarity for the scheme post-contract end in 2024.
